# Transcriptome Changes and Potential Immunotoxicity Analysis in RAW264.7 Macrophages Caused by Bisphenol F

**DOI:** 10.3389/fphar.2022.846562

**Published:** 2022-03-21

**Authors:** Huiling Chen, Yanchao Zhang, Xing Li, Wei Zhang, Haoqi He, Bohai Du, Tianlan Li, Huanwen Tang, Yungang Liu, Li Li, Ming Shi

**Affiliations:** ^1^ Dongguan Key Laboratory of Environmental Medicine, School of Public Health, Guangdong Medical University, Dongguan, China; ^2^ Department of Toxicology, School of Public Health, Southern Medical University, Guangzhou, China; ^3^ Dongguan Liaobu Hospital, Dongguan, China

**Keywords:** bisphenol F, RAW264.7 macrophages, immunotoxicity, apoptosis, transcription

## Abstract

As a viable substitute for bisphenol A (BPA), BPF has been widely used in the plastic industry and daily consumer goods, resulting in its detection in humans at a comparable concentration. Evidence reveals that BPF and BPA may have similar toxic effects due to their similar structures. However, there is less information about BPF and its latent implications on the immune system, which is associated with many disorders. In this study, the *in vitro* toxicity of BPF on RAW264.7 macrophages was explored. The cells were treated with different concentrations of BPF (5, 10, 20, 50, 100, and 200 μM), the cell viability and apoptosis were detected, the gene expression profile was analyzed by whole-transcriptome sequencing, and the mRNA levels were detected by qRT-PCR. The results showed a high concentration of BPF could significantly reduce the survival rate of RAW264.7 macrophages. Although the medium concentration (20–50 μM) of BPF seemed to have no impact on the cell activity of macrophages, it caused the occurrence of apoptosis. The results of differential transcription showed that compared with the control group, 121 genes were upregulated and 82 genes were downregulated in the BPF group. The significantly changed gene functions were mainly concentrated in cell cycle, phagosome, lysosome, and antigen processing and presentation. These findings provide valuable information for correctly understanding the immunotoxicity risk of BPF and may help to improve the hazard identification of bisphenol compounds.

## 1 Introduction

Bisphenol F (BPF, 4,4′- dihydroxy diphenyl-methane), as a viable substitute for bisphenol A (BPA), has been widely used in the manufacture of plastics, epoxy resins, adhesives, water pipes, and food contact materials (Wang et al., 2014). As the output and consumption of BPF increase year by year, it is widely detected in natural water, sediment, sludge, and indoor dust, resulting in a significant increase in the human exposure risk (Zhang et al., 2019). The environmental contamination of BPF could enter into the human body through diet, drinking water, or dermal contact and has been detected in biological samples such as urine, blood, breast milk, and maternal and cord plasma (Niu et al., 2017; Kolatorova et al., 2018; Lehmler et al., 2018; Rocha et al., 2018). It was reported that the concentration of BPF in the human plasma was three times higher than that of BPA (Andra et al., 2015). With the in-depth study of BPF, the adverse effects of BPF, including mutagenicity, genotoxicity, endocrine disorder, developmental toxicity, and immunotoxicity, have attracted people’s attention (Chen et al., 2016; Pelch et al., 2019).

As it is well-known, the immune system is the barrier and the guard of a host body, and macrophages play an important role in both innate and adaptive immunity. Macrophages participate in every stage of the acute immune response, as well as the regulation of tissue homeostasis and the coordination of the tissue repair process (Jain et al., 2019). If the function of macrophages is disturbed, it may lead to diseases, such as cardiovascular and cerebrovascular diseases, metabolic diseases, and cancer (Odegaard and Chawla, 2011; Okabe and Medzhitov, 2016; Ritz et al., 2018; Van den Bossche and Saraber, 2018). Kim et al. found that tetramethyl BPF could activate osteoclast differentiation by activating the MAPK signaling pathway and JNK and p38 in RAW264.7 cells, which had a negative impact on bone remodeling and the stability of the bone environment (Kim et al., 2021). At present, the immunotoxicity of BPA has been confirmed by *in vivo* and *in vitro* experiments (Youn et al., 2002; Rogers et al., 2013). In view of the structural similarity between BPF and BPA, it is reasonable to speculate that BPF may have similar effects on the immune system under environmental or occupational exposure. Qiu et al. found that BPF had its immunotoxicity similar to BPA during the development of zebrafish embryos and larvae, which may pose a risk to the ecosystem and human health (Qiu et al., 2018). Nevertheless, studies on the immunotoxicity of BPF are limited.

RAW264.7 is a type of macrophage derived from murine, which has been widely used to be a good *in vitro* model to evaluate the immunotoxicity of compounds or pollutants (Makene and Pool, 2015; Ye et al., 2015; Zhang et al., 2017). RNA deep sequencing (RNA-seq) is the newly developed high-throughput gene expression quantitative technology, which is utilized to analyze the genome-wide gene expression. RNA-seq can help us to generate an unprecedented global view of the transcriptome and its organization for many species and cell types (Wang et al., 2009). In this study, to investigate the effect of BPF on the RAW264.7 macrophages, cell viability and apoptosis were explored; whole transcriptome sequencing was used to obtain the information on the genome-wide expression changes induced by BPF. The mRNA levels of genes with a significant difference in the cell cycle, phagosome, lysosome, and antigen processing and presentation pathway were detected by qRT-PCR. It is hoped that our research could provide effective information for the potential immunotoxicity of BPF to macrophages.

## 2 Materials and Methods

### 2.1 Cell Culture

The BALB/c-derived RAW264.7 macrophages were a generous gift from Professor Chong Yan (Guangdong Medical University, Dongguan, China). RAW264.7 macrophages were maintained in Dulbecco’s modified Eagle’s medium (DMEM, Grand Island, NY) supplemented with 10% fetal bovine serum (FBS, Bovogen, New Zealand) and incubated at 37°C in a 5% CO_2_ atmosphere. Cells were seeded in a 6-well plate at a density of 5.0 × 10^5^ cells/well and incubated overnight. Then, cells were harvested after 24 h treatment with different concentrations of BPF (CAS No. 620-92-8, purity 98%, Sigma-Aldrich, St. Louis, MO, United States) or BPA (CAS No.80-05-7, purity 97%, Sigma-Aldrich, St. Louis, MO, United States), which was dissolved in 0.1% dimethyl sulfoxide (DMSO; Sigma-Aldrich, St. Louis, MO, United States). Untreated cells (0 μM BPF) were exposed to 0.1% DMSO.

### 2.2 Cell Viability

The cell viability of RAW264.7 macrophages treated with BPF (or BPA) was determined by CCK-8 (Cell Counting Kit-8) (CCK-8, Dojindo Laboratories, Japan). Briefly, RAW264.7 macrophages were seeded into 96-well plates at a density of 1.0 × 10^4^ cells per well. After incubation for 24 h, 0, 1, 5, 10, 20, 50, 100, or 200 μM BPF (or BPA) in DMEM medium was added to 96-well plates and incubated for another 24 h. Then, the CCK-8 solution (10 μl) was added to each well and subsequently cultured for 3 h at 37°C. The absorbance at 450 nm was read by using a microplate reader (ELx808, BioTek Instruments, Inc., Winooski, VT, United States). The cell viability was calculated by the absorbance ratio of the sample well to the control well and expressed as a percentage, assigning the cell viability of the control well as 100%.

### 2.3 LDH Releasing Assay

Lactate dehydrogenase (LDH) activity released in the culture media was measured by the CytoTox 96^®^ non-radioactive cytotoxicity assay (Promega corporation, Wis, United States), according to the manufacturer’s instructions. Absorbance at 490 nm was recorded using a microplate reader. LDH release % = (Experimental LDH Release (OD490)/Maximum LDH Release (OD490)) × 100.

### 2.4 Apoptosis Assay

Apoptosis assay was measured with an Annexin V-FITC/PI Apoptosis Detection Kit (KeyGen Biotech, Nanjing, China), according to the manufacturer’s instructions. In brief, the RAW264.7 macrophages (5 × 10^5^ cell/well) were seeded in a 6-well plate and exposed to BPF at different concentrations. After 24 h treatment, the cells and cell culture medium were collected and centrifuged at 1,000 g for 5 min. The supernatant was discarded, and the cells were suspended with Annexin V-FITC binding buffer and stained with 5 µl Annexin V-FITC and 5 µl PI for 15 min at room temperature. The apoptosis of RAW264.7 macrophages was detected by flow cytometry (FACScantoII System, BD Biosciences, San Jose, CA, United States). All the experiments were conducted in triplicates.

### 2.5 RNA Isolation, cDNA Library Construction, and Sequencing

RAW264.7 macrophages (5 × 10^5^ cell/well) were cultured in 6-well plates and exposed to 20 μM BPF for 24 h. Then, the supernatant was removed, and the cells were washed twice with ice-cold PBS buffer solution. The total RNA was extracted using TRIzol reagent (Life technologies, NY, United States), according to the manufacturer’s protocol. The Bioanalyzer 2100 system (Agilent Technologies, CA, United States) was used to assess the RNA integrity. Samples were sent to Shanghai Jikai Gene Technology Co., Ltd. for whole-transcriptome sequencing analysis. Briefly, a total amount of 1 µg RNA per sample was used as input material for the RNA sample preparations. Sequencing libraries were generated using the NEBNext^®^ UltraTM RNA Library Prep Kit for Illumina^®^ (NEB, Beijing, China), and index codes were added to attribute sequences to each sample. The clustering of the index-coded samples was performed on a cBot cluster generation system using the TruSeq PE Cluster Kit v3-cBot-HS (Illumina), according to the manufacturer’s instructions. After cluster generation, the library preparations were sequenced on an Illumina Novaseq platform, and 150-bp paired-end reads were generated.

### 2.6 Bioinformatics Analysis of Sequencing Results

RNA-seq experiments were performed using the Illumina Novaseq platform. Raw data (raw reads) of fastq format were first processed through in-house perl scripts to obtain clean data (clean reads) by removing reads containing adapter, reads containing ploy-N, and low-quality reads from raw data. The processed reads were mapped to the reference using HISAT2. The mapped reads of each sample were assembled by StringTie (Pertea et al., 2015), and then, FPKM of each gene was calculated based on the length of the gene and read counts mapped to this gene. FPKM, the expected number of fragments per kilobase of transcript sequence per million’s base pairs sequenced, considers the effect of the sequencing depth and gene length for the read counts at the same time, and it is currently the most used method for estimating gene expression levels. Differential expression analysis of two conditions was performed using the edgeR R package. The *p* values were adjusted using the Benjamini & Hochberg method. A corrected *p*-value of 0.05 and |log2 (fold change)| of 0.26 were set as the threshold for significantly differential expressions. The gene ontology (GO) enrichment analysis of differentially expressed genes was implemented by the cluster Profiler R package. The Kyoto Encyclopedia of Genes and Genomes (KEGG) database was used to identify significant pathways of RAW264.7 macrophages altered by BPF. We used the cluster Profiler R package to test the statistical enrichment of differential expression genes in KEGG pathways.

### 2.7 qRT-PCR

The total RNA of RAW264.7 macrophages was extracted with TRIzol reagent, and then, cDNAs were synthesized by using the reverse transcription kit (Takara Bio, Japan), according to the manufacturer’s instructions. The TB Green Real-time PCR kit (Takara Bio, Japan) was used and detected by PikoReal 96 fluorescence quantitative PCR. qRT-PCR amplification was carried out under the following conditions for 40 cycles: 95°C for 30 s, 95°C for 15 s, and 60°C for 30 s. The primers of *H2-T23*, *Sec61a1*, *Sec61b*, *Sec61g*, *Eea1*, *Hspa5*, *Psme2b*, *Psme2*, *Canx*, *Gusb*, *Hexb*, *Atp6v0a1*, *Hyal1*, *Idua*, *Mdm2*, *Cdk1*, *Cdc20*, *Bub1b*, *Dbf4*, *Atp6v0d2*, *Snp23*, *Vamp7*, *Vps34*, *Rab7*, and *β-actin* were synthesized by Shanghai Generay Biotech Co., Ltd. (Shanghai, China), which are listed in Table 1. The relative quantity of mRNA was calculated using the standard 2^−ΔΔCT^ method with β-actin serving as an internal standard.

**TABLE 1:** Primer sequences used for qRT- PCR.

Gene	Forward	Reverse
*Mdm2*	TGT​CTG​TGT​CTA​CCG​AGG​GTG	TCC​AAC​GGA​CTT​TAA​CAA​CTT​CA
*Cdk1*	AGA​AGG​TAC​TTA​CGG​TGT​GGT	GAG​AGA​TTT​CCC​GAA​TTG​CAG​T
*Cdc20*	TTC​GTG​TTC​GAG​AGC​GAT​TTG	ACC​TTG​GAA​CTA​GAT​TTG​CCA​G
*Bub1b*	GAG​GCG​AGT​GAA​GCC​ATG​T	TCC​AGA​GTA​AAA​GCG​GAT​TTC​AG
*Dbf4*	AAT​AAG​ATA​CAG​TGT​CGG​GTC​CC	GTC​CTT​CTG​GAA​ATT​GGG​CTC
*H2-T23*	ACA​GTC​CCG​ACC​CAG​AGT​AG	CCA​CGT​AGC​CGA​CAA​TGA​TGA
*Sec61a1*	GGA​AGT​CAT​CAA​GCC​ATT​CTG​T	GCA​TCC​AGT​AGA​ACG​GGT​CAG
*Sec61b*	TCC​CAG​TGC​TGG​TGA​TGA​GT	GCG​TGT​ACT​TGC​CCC​AAA​T
*Sec61g*	CAG​GTA​ATG​CAG​TTT​GTG​GAG​C	TGG​ATC​AGT​TTC​ACG​AAG​AAG​C
*Eea1*	AAA​CCA​GCT​AAG​GAG​TGA​ACT​TG	GTG​GGT​GTA​GTC​TAG​GTC​TTT​CT
*Gusb*	GGC​TGG​TGA​CCT​ACT​GGA​TTT	GGC​ACT​GGG​AAC​CTG​AAG​T
*Hexb*	CTG​GTG​TCG​CTA​GTG​TCG​C	CAG​GGC​CAT​GAT​GTC​TCT​TG
*Atp6v0a1*	GGA​CCG​ACA​GAG​GAG​GAT​G	GCC​AAA​GTC​AAA​CTC​TTC​TGC​G
*Hyal1*	ACC​TGC​TTC​GCA​TCT​CTA​CTC	GGT​TGG​ATA​CCA​CGG​AAC​CTC
*Idua*	GCT​GAC​CAG​TAC​GAC​CTT​AGT	TAC​GGC​ACC​TAT​GTA​GGC​AAG
*Hspa5*	ACT​TGG​GGA​CCA​CCT​ATT​CCT	ATC​GCC​AAT​CAG​ACG​CTC​C
*Psme2*	GAG​AAG​CCC​GAA​AAC​AGG​TG	AGA​GCT​GAC​TCA​GGG​ATA​TGA​TT
*Canx*	ATG​GAA​GGG​AAG​TGG​TTA​CTG​T	GCT​TTG​TAG​GTG​ACC​TTT​GGA​G
*Atp6v0d2*	CAG​AGC​TGT​ACT​TCA​ATG​TGG​AC	AGG​TCT​CAC​ACT​GCA​CTA​GGT
*Snap23*	CGG​GCT​CAC​CAG​GTT​ACT​G	GGC​TAA​ACC​CAG​GAT​TCT​CCT​T
*Rab7*	AGG​CTT​GGT​GCT​ACA​GGA​AAA	CTT​GGC​CCG​GTC​ATT​CTT​GT
*Vps34*	CCT​GGA​CAT​CAA​CGT​GCA​G	TGT​CTC​TTG​GTA​TAG​CCC​AGA​AA
*Vamp7*	GAC​AAC​TTA​CGG​TTC​CAA​GAG​CA	TCT​CCA​CGT​TGA​GCA​ACT​AAA​TC
*β-actin*	GGC​TGT​ATT​CCC​CTC​CAT​CG	CCA​GTT​GGT​AAC​AAT​GCC​ATG​T

### 2.8 Statistical Analysis

All data were analyzed by GraphPad Prism version 8.0.2. A one-way analysis of variance (ANOVA) with Duncan’s multiple range tests was performed for each response variable. Significance was determined at *p* < 0.05.

## 3 Results

### 3.1 Cytotoxicity of BPF

**FIGURE 1:**
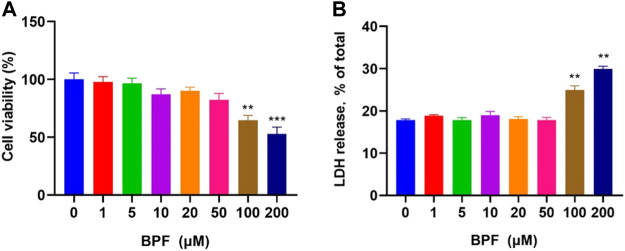
Cytotoxicity induced by BPF on RAW264.7 macrophages: **(A)** CCK-8 assay. **(B)** LDH release assay (*n* = 5). After calculation, the 50% inhibitory concentration (IC_50_) of BPF was 248.60 μM. Data are expressed as means ± SEM. **p < 0.01 and ***p < 0.001 are compared with the control group (0 μM).

The cytotoxicity of BPF on RAW264.7 macrophages was measured by CCK-8 and LDH releasing assay after 24 h treatment. The result of the CCK-8 assay showed that when the concentration of BPF was lower than 100 μM, the viability of macrophages was not significantly affected (*p* > 0.05). When the BPF concentration was 100 and 200 μM, it could significantly inhibit the viability of macrophages. The inhibition rate on cell viability of 200 μM BPF was close to 50% compared to the control group (Figure 1A). Under normal conditions, LDH exists in the cytoplasm. When the integrity of the phospholipid bilayer structure of the cell membrane is destroyed, LDH will leak into the culture medium through the incomplete cell membrane. Hence, the degree of cell damage can be reflected by detecting the LDH activity in the cell culture supernatant. The result of LDH releasing assay presented that BPF significantly promoted the release rate of LDH at the concentration of 100 and 200 μM (Figure 1B), which was consistent with the result of CCK-8 assay. The aforementioned results showed that there was no obvious cytotoxicity of BPF on RAW264.7 macrophages when the concentration was lower than 100 μM, but high concentrations of BPF could significantly reduce the survival rate of RAW264.7 macrophages. Furthermore, the cytotoxicity of BPA was detected by CCK-8 and LDH assays (Supplementary Figures S1A,B). It is proved that the cytotoxicity of BPF is lower than that of BPA on RAW 264.7 macrophages.

### 3.2 The Effect of BPF on RAW264.7 Macrophage Apoptosis

**FIGURE 2:**
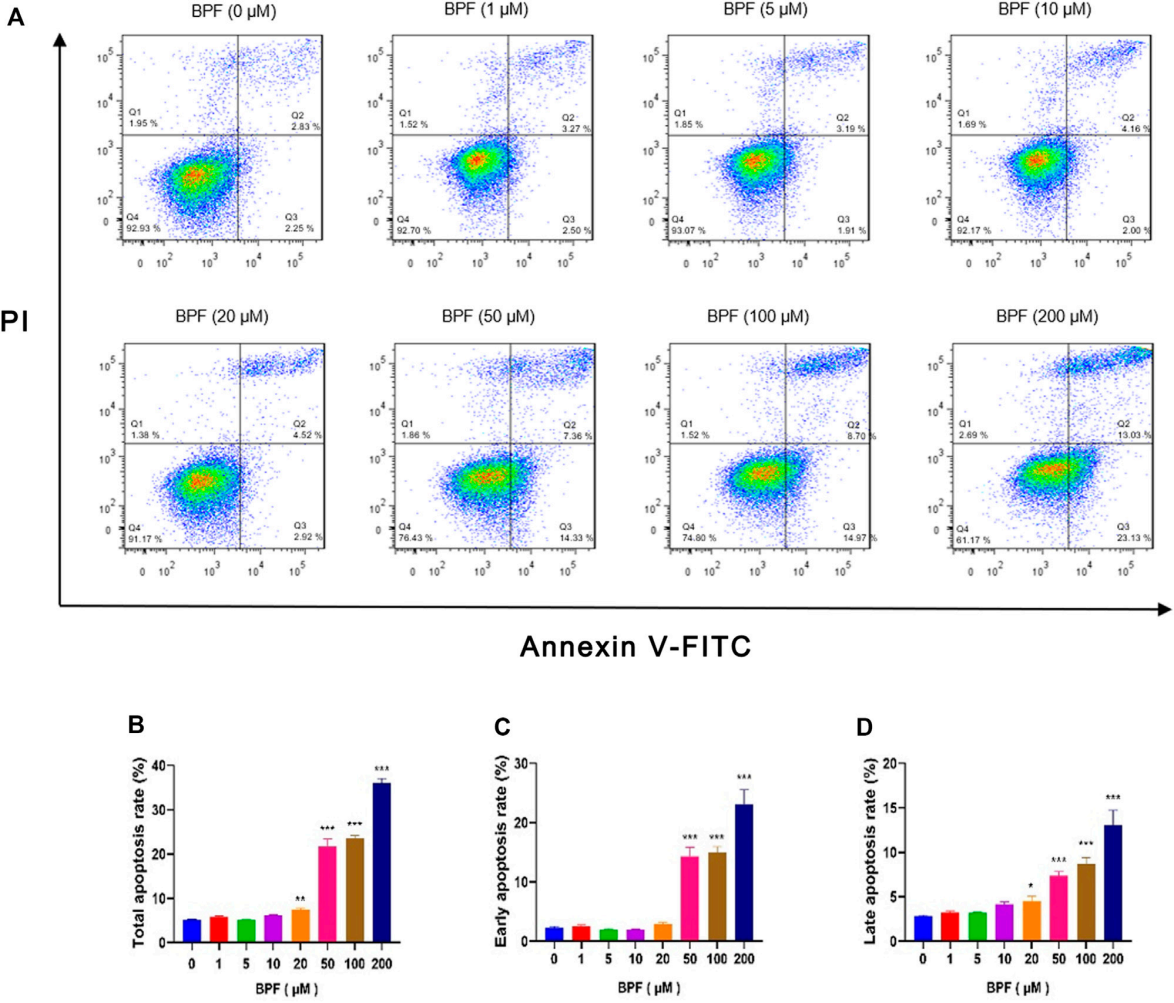
Apoptosis induced by BPF on RAW264.7 macrophages. **(A)** Flow cytometry was used to analyze the cell apoptosis status of RAW264.7 (*n* = 3). **(B)** The total apoptosis rates. **(C)** The early apoptosis rates. **(D)** The late apoptosis rates. Data are expressed as means ± SEM. *p < 0.05, **p < 0.01, and ***p < 0.001 are compared with the control group (0 μM).

In this study, flow cytometric measurement (Annexin V/FITC and PI double staining) was used to quantify the extent of apoptosis of RAW264.7 macrophages after BPF exposure. In normal cells, phosphatidylserine (PS) is located on the inner side of the lipid bilayer of the cell membrane, and the cell membrane is intact. When cells undergo early apoptosis, the cell membrane remains intact, while PS everts to the side of the cell membrane and binds with annexin V-FITC with high affinity to emit green fluorescence. In the late stage of apoptosis, the cell membrane structure is damaged. Propidium iodide (PI) enters the cell through the damaged cell membrane, and the cells emit red fluorescence and green fluorescence at the same time. The proportion of cells with early apoptosis and late apoptosis was analyzed by detecting the signals of annexin V-FITC (green fluorescence) and PI (red fluorescence) simultaneously. The total apoptosis rate is the sum of the early apoptosis rate and late apoptosis rate. It was found that the BPF exposure for 24 h triggered macrophage apoptosis in a concentration-dependent manner (Figure 2A). BPF concentrations at 20, 50, 100, and 200 μM induced approximately 7.40, 21.69, 23.66, and 36.16% total apoptosis rate (Figure 2B) and 4.52, 7.36, 8.70, and 13.03% late apoptosis rate, respectively (*p* < 0.05) (Figure 2D). Moreover, treatment with 50, 100, and 200 μM BPF induced approximately 14.33, 14.97, and 23.13% early apoptosis rate, respectively (*p* < 0.001) (Figure 2C). These results showed that the apoptosis events of RAW264.7 macrophages treated with non-cytotoxic doses (20–50 μM) of BPF observed in CCK-8 assay were significantly increased. It suggested that although the medium dose of BPF exposure seemed to have no impact on the cell activity of macrophages, it caused the occurrence of apoptosis.

### 3.3 Global Analysis of Gene Expressions by RNA-Seq

**FIGURE 3:**
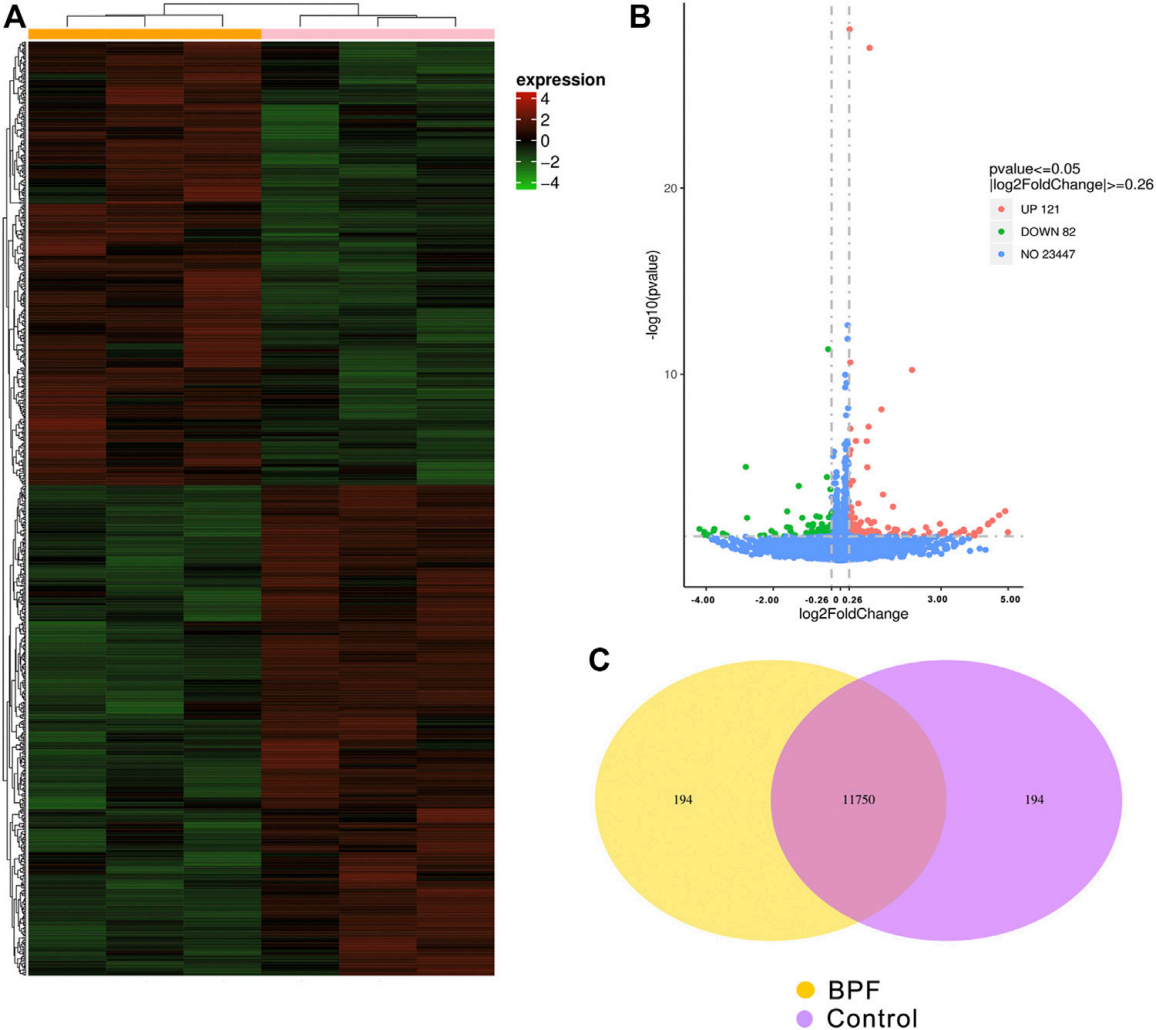
Differential expression patterns of mRNAs between control and BPF-exposed RAW264.7 macrophages. **(A)** The cluster heat map. **(B)** The volcano map of all the sequenced genes from the samples (*p* < 0.05 and |log2 (fold change)| > 0.26). **(C)** Venn diagram of co-expressed genes between control and BPF-exposed RAW264.7 macrophages.

RNA-seq was applied to identify gene expression changes induced by BPF treatment in RAW264.7 macrophages. The heat map (Figure 3A) showed the expression amount of differentially expressed genes between the BPF group and control group. The genes with high expression (red) and low expression (green) were distributed in the opposite direction, indicating that BPF treatment induced significant differences in the gene expression. The volcano map (Figure 3B) revealed that there were 203 differentially expressed genes (DEGs) between the BPF group and control group. Of these genes, 121 genes were upregulated, and 82 genes were downregulated by BPF. The Venn diagram (Figure 3C) could show the overlap of different genes between the BPF group and control group. There were 11,750 genes expressed both in the control group and BPF group, and 194 genes were expressed only in the BPF group.

### 3.4 Functional Enrichment Analysis of Differentially Expressed Genes

**FIGURE 4:**
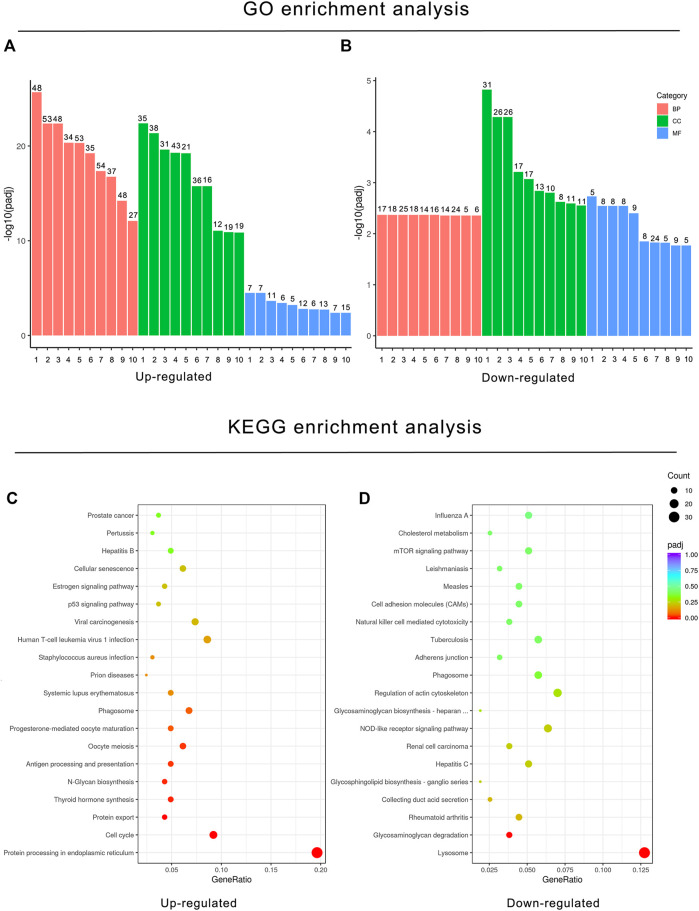
Functional enrichment analysis of DEGs based on the GO annotation category and KEGG pathway category. GO enrichment analysis histogram. *X*-axis: the serial number of GO Term (see Supplementary Tables S1 and S2 for function description corresponding to GO enrichment analysis); *Y*-axis: the significant difference of GO enrichment between the control group and BPF treatment group (−log10 *p* value). **(A)** GO enrichment analysis histogram of upregulated DEGs. **(B)** GO enrichment analysis histogram of downregulated DEGs. **(C)** KEGG bubble charts of upregulated DEGs **(C)** and downregulated DEGs **(D)**. The size of dots represents the number of genes annotated to GO enrichment or the KEGG pathway, and the color from red to purple represents the significance of enrichment.

To understand the implications of these differentially expressed genes and clarify the potential regulatory mechanisms on BPF-treated RAW264.7 macrophages, functional enrichment analysis based on the GO annotation category and KEGG pathway category was performed. The GO annotation category is a comprehensive database describing the gene function, which can be divided into the biological process (BP), cellular component (CC), and molecular function (MF). The adjusted *p* value less than 0.05 was taken as the threshold value for significant enrichment of GO function, and the most significant 30 terms were shown in the histogram. In this study, the upregulated genes after BPF treatment were enriched in mitosis, cell cycle, chromosome segregation, and so on. However, the downregulated genes were enriched in the immune response and ATPase activity (Figures 4A,B; Supplementary Tables S1 and S2). KEGG is a database resource to understand the advanced functions and utility of biological systems (such as cells, organism, and ecosystems) by using molecular level information such as large-scale molecular data sets generated by genome sequencing and other high-throughput experimental technologies. KEGG pathway enrichment analysis takes the adjusted *p* value < 0.05 as the threshold of significant enrichment. When RAW264.7 macrophages were treated with BPF for 24 h, the most significant 20 terms of the KEGG pathway were shown in the bubble charts (Figures 4C,D). The upregulated genes were enriched in protein processing in the endoplasmic reticulum, protein export, cell cycle, and so on. However, the downregulated genes were enriched in lysosome, glycosaminoglycan degradation, rheumatoid arthritis, and so on.

### 3.5 Effect of BPF on Immune Function-Related Pathways

In KEGG enrichment analysis, the following terms were significantly enriched in our gene list: protein processing in the endoplasmic reticulum (mmu04141), lysosome (mmu04142), phagosome (mmu04145), cell cycle (mmu04110), antigen processing and presentation (mmu04612), N-Glycan biosynthesis (mmu00510), protein export (mmu03060), and glycosaminoglycan degradation (mmu00531), which are exhibited in Table 2. The upregulated and downregulated DEGs in the top eight most significant results of KEGG enrichment are shown in Table 3. Terms including lysosome (mmu04142), phagosome (mmu04145), and antigen processing and presentation (mmu04612) provided clues that BPF may have effect on immune function of RAW264.7 macrophages.

**TABLE 2:** Significant KEGG pathways from differentially expressed genes.

Terms	*p* Adj	Transcriptome count	Fold enrichment
mmu04141: Protein processing in endoplasmic reticulum	<0.001	34	3.73
mmu04142: Lysosome	<0.01	20	2.93
mmu04145: Phagosome	<0.05	20	2.48
mmu04110: Cell cycle	<0.05	16	2.30
mmu04612: Antigen processing and presentation	<0.05	12	3.19
mmu00510: N-Glycan biosynthesis	<0.05	10	3.86
mmu03060: Protein export	<0.05	7	4.76
mmu00531: Glycosaminoglycan degradation	<0.05	6	6.00

**TABLE 3:** Upregulated and downregulated DEGs in the top eight most significant results of KEGG enrichment.

Description	Count	Upregulated	Downregulated	Gene name	
				Upregulated	Downregulated
Protein processing in the endoplasmic reticulum	34	32	2	Hspa5/Ssr1/Hyou1/Hsp90b1/P4hb/Pdia6/Xbp1/Sec24d/Selenos/Ssr3/Rpn1/Sec61a1/Stt3a/Sec61b/Canx/Ssr2/Sec61g/Pdia4/Dnajc3/Tram1/Txndc5/Dnajb11/Syvn1/Rpn2Sec23b/Sec24c/Ddost/Pdia3/Calr/Lman1/Herpud1/Edem2	Man1c1/Atxn3
Lysosome	20	0	20	/	Gusb/Hexa/Gns/Ctsd/Igf2r/Ctsa/Hexb/Ap1g2/Atp6v0a1/Gaa/Litaf/Hyal1/Ap1m1/Ctsb/Gga2/Laptm5/Ctsz/Gga1/Atp6v0d1/Idua
Phagosome	20	11	9	Sec61a1/Sec61b/Canx/Sec61g/Eea1/Ncf2/Tubb4b/Cd36/H2-Q6/Calr/H2-Eb1	H2-T23/Atp6v1c1/Coro1a/Atp6v0a1/Itgb2/Colec12/Itgb5/Atp6v0d1/Atp6v1a
Cell cycle	16	15	1	Cdk1/Cdc20/Ttk/Ccnb1/Bub1/Tfdp2/Bub1b/Cdc25b/Rad21/Ccna2/Smc1a/Mad2l1/Dbf4/Ccnb2/Mdm2	Tgfb1
Antigen processing and presentation	12	8	4	Hspa5/Psme2/Canx/H2-Q6/Pdia3/Calr/B2m/H2-Eb1	Psme2b/H2-T23/Ctsb/Tapbp
N-Glycan biosynthesis	10	7	3	Rpn1/Stt3a/Alg2/Mgat2/Rpn2/Ddost/Alg13	Man1c1/Man2a2/Dpm3
Protein export	7	7	0	Hspa5/Sec61a1/Spcs2/Sec61b/Sec61g/Spcs3/Srp72	/
Glycosaminoglycan degradation	6	0	6	/	Gusb/Hexa/Gns/Hexb/Hyal1/Idua

### 3.6 Effects of BPF on the Cell Cycle, Phagosome, Lysosome, and Antigen Processing and Presentation Pathway

**FIGURE 5:**
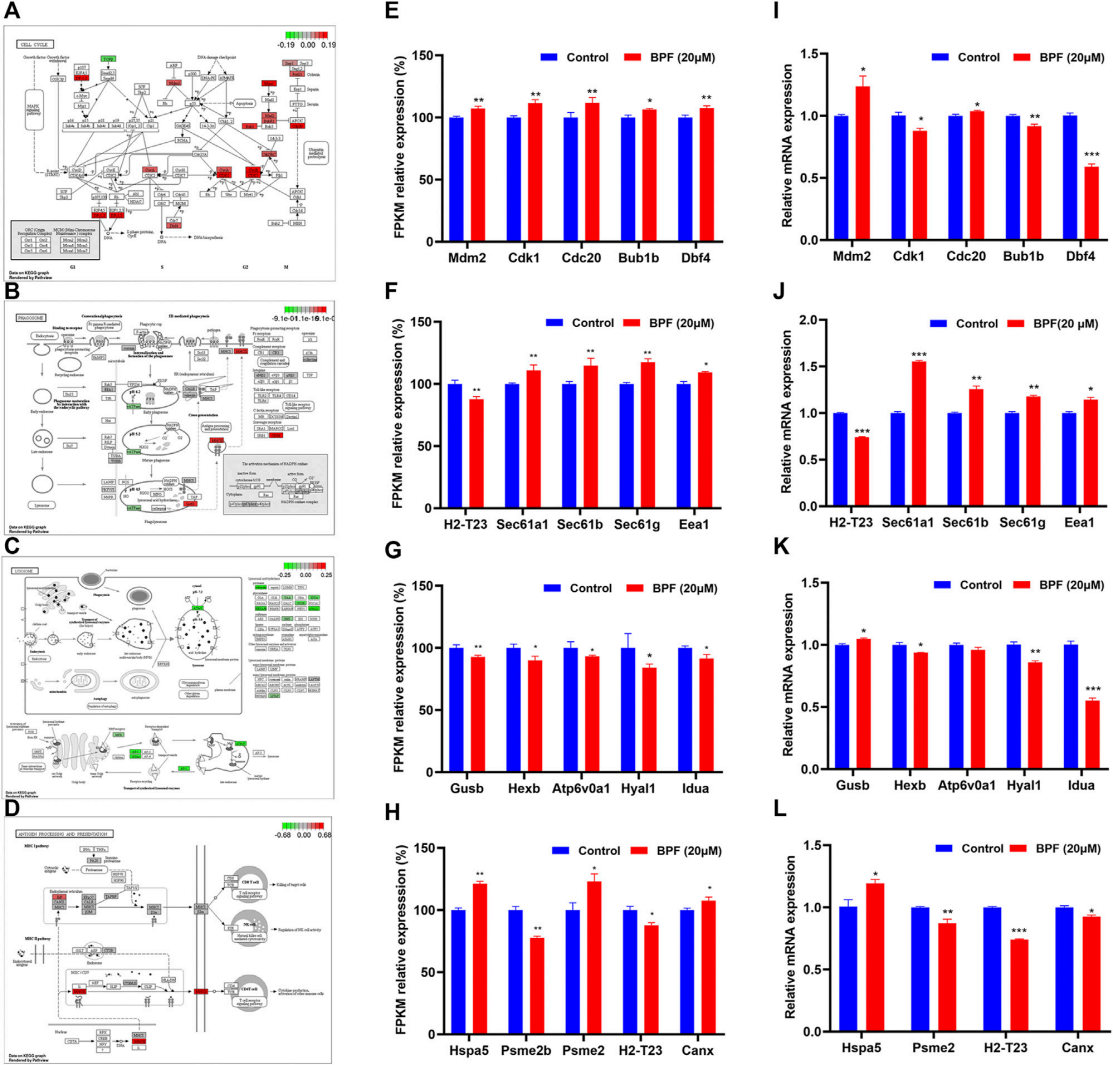
Results of the effects of BPF on the cell cycle, phagosome, lysosome, and antigen processing and presentation pathway. **(A–D)** Schematic diagram of the KEGG signaling pathway of cell cycle, phagosome, lysosome, and antigen processing and presentation pathway induced by BPF, respectively. Red and green symbols represent upregulated and downregulated gene expressions. **(E–H)** FPKM/relative expression levels of the top five genes with significant difference in the aforementioned KEGG signaling pathway. **(I–L)** The mRNA levels of the top five genes in the aforementioned KEGG signaling pathway re-confirmed by qRT-PCR. Data are expressed as means ± SEM. *p < 0.05, **p < 0.01, and ***p < 0.001 are compared with the control group (0 μM).

As shown in Figure 5, the influence of BPF on the cell cycle, phagosome, lysosome, and antigen processing and presentation pathway was depicted by using the path view package in R software according to the results from cluster analyses (Figures 5A–D), and the FPKM relative expressions of the top five genes with significant differences in these four pathways are represented by a histogram. BPF upregulated the expressions of *Mam2*, *Cdk1*, *Cdc20*, *Bub1b*, and *Dbf4* of the cell cycle pathway (Figure 5E), upregulated the expressions of *Sec61a1*, *Sec61b*, *Sec61g*, and *Eea1*, and downregulated *H2-T23* in the phagosome pathway (Figure 5F). At the same time, BPF downregulated the expressions of *Gusb*, *Hexb*, *Atp6v0a1*, *Hyal1*, and *Idua* of the lysosome pathway (Figure 5G). In the antigen processing and presentation pathway, the expressions of *Hspa5 and Psme2* were upregulated, and the expressions of *Psme2b and H2-T23* were downregulated (Figure 5H).

The top five genes with significant differences in these four pathways were re-confirmed by qRT-PCR. The results showed that BPF upregulated the expressions of *Mam2* and *Cdc20*, downregulated those of *Cdk1*, *Bub1b*, and *Dbf4* of the cell cycle pathway (Figure 5I), upregulated the expressions of *Sec61a1*, *Sec61b*, *Sec61g*, and *Eea1*, and downregulated *H2-T23* in the phagosome pathway (Figure 5J). In addition, BPF downregulated the expressions of *Hexb*, *Hyal1*, and *Idua* and upregulated the expression of *Gusb* in the lysosome pathway (Figure 5K). In the antigen processing and presentation pathway, the expression of *Hspa5* was upregulated, and the expressions of *Psme2*, *H2-T23*, and *Canx* were downregulated (Figure 5L). Although the expression of a few genes differed from the results of RNA-seq, these results still suggested that BPF could affect the cell cycle, phagocytosis, and antigen processing and presentation of macrophages.

The fusion of phagosome and lysosome is the main driving factor of phagosome maturation and is targeted by several adaptive intracellular pathogens. The damage of this process will have a significant impact on microbial infection, tissue inflammation, adaptive immunity, and diseases. Given the importance of phagosome-lysosome fusion to phagocyte function, the genes related to the phagosome-lysosome fusion (*Atp6v0d2*, *Snap23*, *Rab7*, *Vps34*, and *Vamp7*) were selected and detected by qRT-PCR. The expressions of *Atp6v0d2*, *Vamp7*, and *Vsp34* were significantly downregulated, while that of *Snap23* was upregulated by BPF (Supplementary Figure S2). It suggested that BPF may affect the phagosome-lysosome fusion of RAW264.7 macrophages.

## 4 Discussion

Although the immunotoxicity of environmental pollutants has drawn much attention, there are few studies on the effect of BPF on the immune system. Švajger et al. reported that 50 μM of BPF and BPA significantly decreased the endocytotic capacity of immature monocyte-derived dendritic cells (iMDDCs) (Švajger et al., 2016). It suggested BPF and BPA may have similar immunotoxicity mechanisms. Yann et al. reported that BPF and BPA significantly increased IL-17 production in mouse T cells but not in human T lymphocytes at low and environmentally relevant concentrations. However, BPF, not BPA, could increase IL-17 secretion in mouse naive T cells undergoing Th17 differentiation *in vitro* (Malaisé et al., 2020a). In addition, Yann et al. reported that pregnant C3H/HeN mice exposed to 5 or 50 mg/kg body weight of BPA, or BPF daily from gestation day 15 to weaning, exhibited altered immune profiles. Only exposure to the high dose of BPA decreased IgA levels in the feces of the adult offspring (Malaisé et al., 2020b). These studies suggest that the immunotoxicity mechanisms of BPF and BPA are not identical. In our previous study, we found BPF induced M1 polarization and promoted the secretion of pro-inflammatory cytokines of RAW264.7 macrophages, which suggested that BPF enable affecting the immune function of macrophages (Shi et al., 2020; Zhang et al., 2021). The potential molecular mechanisms underlying these effects of BPF remain largely obscure. It is urgent to understand the potential mechanisms of BPF. Nowadays, transcriptomics analysis is widely considered as a novel and valuable strategy to conduct mechanistic studies (Tasic et al., 2016; Lee and Park, 2019). In this study, we found that high doses (100 and 200 μM) of BPF exposure significantly reduced the cell viability, and BPF induced the apoptosis at 20 μM and higher concentrations. On this basis, RNA-sequencing of RAW264.7 macrophages after treatment with 20 μM BPF was performed, and it was noticed that BPF has a significant effect on the cell cycle and immune-related functions such as lysosome transport and catabolism, phagosome transport and catabolism, and antigen processing and presentation.

Under the homeostatic physiological conditions, macrophages are a ubiquitous cellular component present in all tissues and body compartments (Ginhoux and Guilliams, 2016). Macrophages are highly plastic in the innate immune system and play an important role in resisting microorganisms and a variety of diseases. In addition, macrophages have many non-immunological effects during development, such as functions in morphogenesis of other tissues (neurons and bone), angiogenesis, and adipogenesis (Pollard, 2009). However, the death of macrophages, especially apoptosis, could aggravate the progress of certain diseases, such as viral infection, tuberculosis, atherosclerotic plaque formation, inflammation, and sepsis (Robinson et al., 2019). Exposure to environmental pollutants may induce apoptosis of macrophages. In the present study, only exposure to higher concentrations of BPF (100–200 μM) significantly reduced the cell viability but BPF could induce apoptosis of RAW264.7 macrophages at the non-cytotoxic concentrations (20–50 μM). This indicated that a certain BPF exposure seemed to have no impact on macrophage viability but caused the occurrence of apoptosis. These results suggested that exposure to BPF may increase the risk of diseases by inducing apoptosis and cell death of macrophages.

As lower concentrations of BPF could induce the late apoptosis while having no effect on the cell viability in RAW264.7 macrophages, 20 μM of BPF was selected for RNA-seq. From mRNA transcriptome sequencing results, 203 DEGs were obtained after BPF treatment, suggesting that BPF exposure induced adverse biological effects on RAW264.7 macrophages. GO enrichment analysis of all DEGs suggested that the cell division processes and immune response of the RAW264.7 macrophages were significantly disturbed by BPF treatment. In KEGG enrichment analysis, the cell cycle pathway and the immune-related pathways including phagosome, lysosome, and antigen processing and presentation pathway were significantly regulated. The aforementioned results suggested that the immune system of RAW264.7 macrophages was also severely affected after exposure to BPF.

The anaphase-promoting complex/cyclosome (APC/C) and cyclin-dependent kinase 1 (Cdk1) are two of many important cell cycle regulators. They control the cell cycle through ubiquitination and phosphorylation, respectively. APC/C performs spatiotemporal regulation through a variety of mechanisms, including phosphorylation, the interaction of structurally related co-activators Cdc20 and Cdh1, loading different E2 ubiquitin-binding enzymes, binding to inhibitors, and different affinities to various substrates (Yamano, 2019). In this study, the results of KEGG enrichment analysis showed that the expressions of *Cdk1* and *Cdc20* in RAW264.7 macrophages were upregulated after exposure to BPF for 24 h. *Mdm2*-encoded protein can promote tumor formation by targeting tumor suppressor proteins, such as p53, for proteasomal degradation. Meanwhile, the gene of *Bub1b* was the essential component of the mitotic checkpoint, which may play a role for tumor suppression. We found the expression of *Mdm2* was upregulated, and the expression of *Bub1b* was downregulated after treatment with BPF for 24 h by qRT-PCR. These results suggested that BPF may lead to the disorder of the macrophage cell cycle.

Phagocytosis, a symbol in host defense, is the fundamental process of macrophages to ingest and eliminate pathogens, which requires dynamic changes in plasma membrane fusion and fission. Formation of phagosomes is the key to phagocytosis. We found that the genes of *Sec61a1*, *Sec61b*, *Sec61g*, and *Eea1* were influenced with BPF treatment in RAW264.7 macrophages, which were involved in the formation of phagosomes. Lysosomes are one of kingpins in macrophages. When tissues are infected, macrophages phagocytize and internalize the pathogen into phagosomes and then fuse with lysosomes to obtain an acidic environment that kills and degrades the closed microorganisms (Fairn and Grinstein, 2012; Levin et al., 2016; Pauwels et al., 2017). More than 60 lysosomal hydrolases have been identified to decompose proteins, lipids, nucleic acids, and carbohydrates (Appelqvist et al., 2013; Xu and Ren, 2015). In this study, BPF influenced the expressions of *Gusb*, *Hexb*, and *Idua* that encode lysosomal hydrolases in RAW264.7 macrophages, which suggested that BPF may affect the production of lysosome hydrolases. The activities of lysosomal hydrolases require an acidic environment, which is achieved by the proton pump activity of V-ATPase. Atp6v0d2, together with Atp6v1d and Atp6v1f, forms a central stalk of the V-ATPase. Cotter, K et al. identified a non-redundant role for Atp6v0d2 in the formation of the autolysosome that enables efficient clearing of damaged organelles and ingested bacteria, which in turn limits inflammation (Cotter et al., 2015). Another key process by which macrophages play phagocytosis is the fusion of phagolysosomes. In eukaryotic cells, biological membrane fusion is generally executed by fusogenic-soluble N-ethylmaleimide–sensitive factor (NSF) attachment protein (SNAP) receptors (SNAREs) (Chen and Scheller, 2001). Snap23 and vesicle-associated membrane protein 7 (Vamp7) are ubiquitously expressed SNARE proteins that regulate phagosome formation and maturation in macrophages (Sakurai et al., 2012; Aksoy et al., 2018). The earlier studies showed that inhibition of Vps34 (class III PI3Ks) in macrophages leads to the phagolysosome fusion defect, which hinders phagocytes from acquiring late endosomal/lysosomal products (Vieira et al., 2001). Rab7, a small GTPase, is momentous for the fusion between late-stage phagosomes and lysosomes, which will affect phagocytes to remove pathogens and apoptotic cells (Via et al., 1997; Harrison et al., 2003). Our data showed that *Atp6v0d2*, *Vamp7*, and *Vsp34* were significantly downregulated while *Snap23* was upregulated by BPF. These results suggested that BPF may affect the phagosome-lysosome fusion of RAW264.7 macrophages.

Antigen processing and presentation is another major immune function of macrophages. Macrophages recognize foreign antigens in class I and II major histocompatibility complexes (MHCs) and present them to T cells, which recognize MHC-antigen complexes through their T-cell receptors (Guerriero, 2019). It is well known that CD8^+^ T cells usually recognize peptides on MHC I molecules, while CD4^+^ T cells recognize peptides (swallowed by APC and digested into antigen peptides in phagocytosis/lysosome) on MHC II molecules (Inaba and Inaba, 2005). The genes of *Hspa5*, *Psme2*, *H2-T23*, and *Canx* were important for MHC I antigen processing and presentation. In this study, the expression of *Hspa5* was upregulated, and those of *Psme2*, *H2-T23*, and *Canx* were downregulated by BPF. These suggested that BPF might affect antigen processing and presentation in macrophages.

## 5 Conclusion

In total, we found that a higher dose (100 and 200 μM) of BPF exposure significantly altered the cell viability and induced apoptosis at 20 μM and higher concentrations on RAW264.7 macrophages. Furthermore, this study unveiled the potential immunotoxicity of BPF on macrophages from a transcriptomics perspective. In this current study, 121 upregulated genes and 82 downregulated genes were identified. GO and KEGG pathway analysis demonstrated that these differentially expressed genes were mainly clustered in cell division processes and immune-related biological processes. Moreover, we verified the sequencing results by qRT-PCR. The results were basically consistent with the sequencing results, indicating that BPF has potential immunotoxic effects on macrophages. Collectively, our data indicate that BPF may exert harmful effects *via* regulation of these functional pathways and related biological processes. Nevertheless, further studies are still needed to verify whether these differentially expressed genes have a regulatory function on BPF-induced macrophage immunotoxicity.

## Data Availability

The datasets presented in this study can be found in online repositories. The names of the repository/repositories and accession number(s) can be found below: https://www.ncbi.nlm.nih.gov/sra; SRR17509627, SRR17509626, SRR17509625, SRR17509624, SRR17509623, and SRR17509622.
