# Supplementary material for: Transcriptome Changes and Potential Immunotoxicity Analysis in RAW264.7 Macrophages Caused by Bisphenol F
Source: Front Pharmacol. 2022 Mar 21;13:846562. doi: 10.3389/fphar.2022.846562 (PMC8978606; doi:10.3389/fphar.2022.846562)
Supplement: Supplementary file 1 [file DataSheet1.docx]

**Supplementary materials**

Transcriptome changes and Potential Immunotoxicity Analysis in RAW264.7 macrophages caused by Bisphenol F

Huiling Chen^1, †^, Yanchao Zhang^1, †^, Xing Li^1^, Wei Zhang^1^, Haoqi He^1^, Bohai Du^1^, Tianlan Li^1^, Huanwen Tang^1^, Yungang Liu^2^, Li Li^1,*^, Ming Shi^1, 3*^

^1^ Dongguan Key Laboratory of Environmental Medicine, School of Public Health, Guangdong Medical University, Dongguan, 523808, Guangdong Province, China

^2^ Department of Toxicology, School of Public Health, Southern Medical University, Guangzhou, 510515, Guangdong Province, China

^3^ Dongguan Liaobu Hospital, Dongguan, 523808, Guangdong Province, China

*** Correspondence:**Dr. Li Li
Email: lily2017@gdmu.edu.cn

Dr. Ming Shi
Email: shiming@gdmu.edu.cn

^†^ These authors have contributed equally to this work and share first authorship.


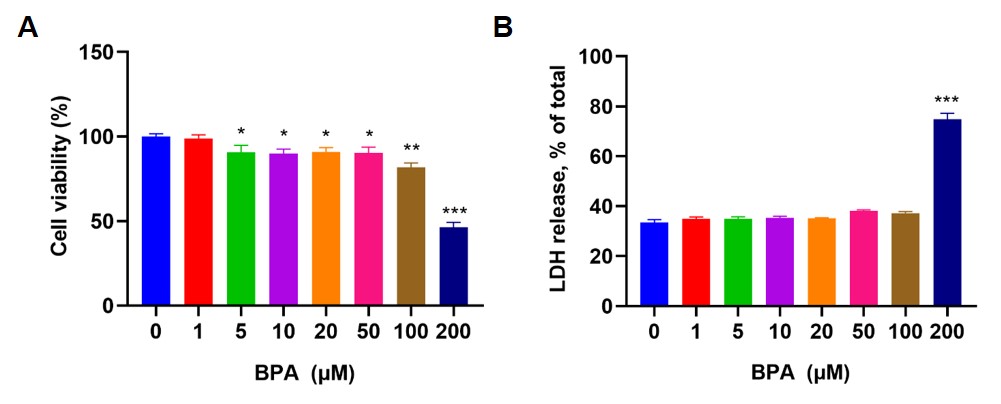


**Figure S1.** Cytotoxicity induced by BPA on RAW264.7 macrophages: (A) CCK-8 assay. (B) LDH release assay (n=5). After calculation, the 50% inhibitory concentration (IC_50_) of BPA was 152.67 μM. Data are expressed as means ± SEM. ^*^*P* < 0.05, ^**^*P* < 0.01, ^***^*P* < 0.001 compared with the control group (0 μM).


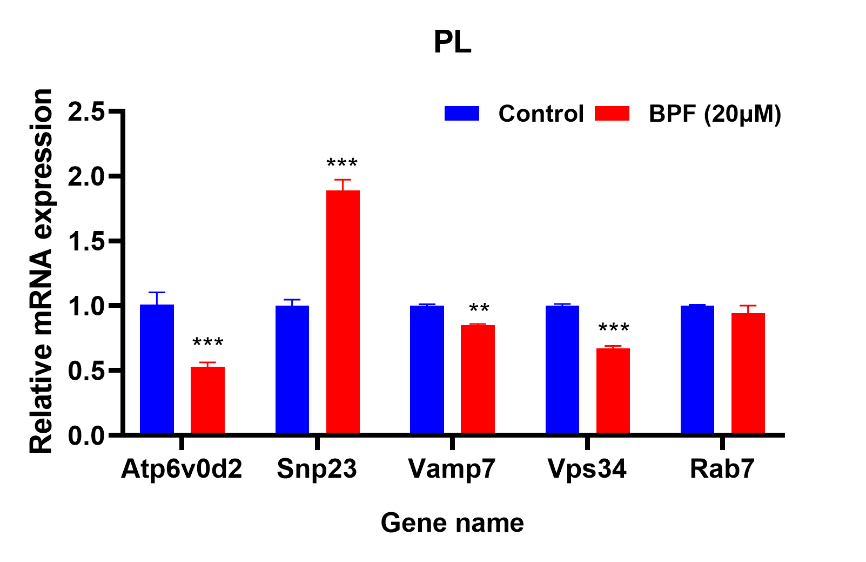


**Figure S2.** The qRT-PCR was used to demonstrate genes changes of the phagosome-lysosome fusion in macrophages after BPF treatment. Data are expressed as means ± SEM. *^*^P* < 0.05, *^**^P* < 0.01, *^***^P* < 0.001 compared with the control group (0 μM).

Table S1. Function description corresponding to GO enrichment analysis of up-regulated DEGs

|  |  | Terms | | |
| --- | --- | --- | --- | --- |
|  |  | BP | CC | MF |
| Up-regulated | 1 | mitotic nuclear division | condensed chromosome | protein disulfide isomerase activity |
|  | 2 | nuclear division | chromosome, centromeric region | intramolecular oxidoreductase activity, transposing S-S bonds |
|  | 3 | chromosome segregation | kinetochore | microtubule motor activity |
|  | 4 | mitotic sister chromatid segregation | chromosomal region | histone kinase activity |
|  | 5 | organelle fission | condensed chromosome, centromeric region | peptide disulfide oxidoreductase activity |
|  | 6 | sister chromatid segregation | spindle | motor activity |
|  | 7 | regulation of cell cycle process | condensed chromosome kinetochore | misfolded protein binding |
|  | 8 | nuclear chromosome segregation | condensed nuclear chromosome, centromeric region | isomerase activity |
|  | 9 | regulation of mitotic cell cycle | condensed nuclear chromosome | intramolecular oxidoreductase activity |
|  | 10 | spindle organization | mitotic spindle | microtubule binding |

Table S2. Function description corresponding to GO enrichment analysis of down-regulated

|  |  | Terms | | |
| --- | --- | --- | --- | --- |
|  |  | BP | CC | MF |
| Down-regulated | 1 | cell activation involved in immune response | vacuole | 2'−5'−oligoadenylate synthetase activity |
|  | 2 | immune response−activating signal transduction | lytic vacuole | cation−transporting ATPase activity |
|  | 3 | positive regulation of immune response | lysosome | active ion transmembrane transporter activity |
|  | 4 | immune response−regulating signaling pathway | adherens junction | ATPase coupled ion transmembrane transporter activity |
|  | 5 | myeloid leukocyte activation | anchoring junction | hydrolase activity, hydrolyzing O-glycosyl compounds |
|  | 6 | leukocyte activation involved in immune response | cell cortex part | exopeptidase activity |
|  | 7 | phagocytosis | cortical cytoskeleton | GTPase binding |
|  | 8 | positive regulation of cellular component movement | cortical actin cytoskeleton | proteoglycan binding |
|  | 9 | regulation of ribonuclease activity | focal adhesion | hydrolase activity, acting on glycosyl bonds |
|  | 10 | energy coupled proton transmembrane transport, against electrochemical gradient | cell−substrate adherens junction | ATPase activity, coupled to transmembrane movement of ions, rotational mechanism |
